# So Many Choices: A Guide to Selecting Among Methods to Adjust for Observed Confounders

**DOI:** 10.1002/sim.10336

**Published:** 2025-02-13

**Authors:** Luke Keele, Richard Grieve

**Affiliations:** ^1^ Dept. of Surgery University of Pennsylvania Pennsylvania USA; ^2^ Department of Health Services Research and Policy London School of Hygiene and Tropical Medicine London UK

**Keywords:** confounders, machine learning, statistical adjustment

## Abstract

Non‐randomised studies (NRS) typically assume that there are no differences in unobserved baseline characteristics between the treatment groups under comparison. Traditionally regression models have been deployed to estimate treatment effects adjusting for observed confounders but can lead to biased estimates if the model is missspecified, by making incorrect functional form assumptions. A multitude of alternative methods have been developed which can reduce the risk of bias due to model misspecification. Investigators can now choose between many forms of matching, weighting, doubly robust, and machine learning methods. We review key concepts related to functional form assumptions and how those can contribute to bias from model misspecification. We then categorize the three frameworks for modeling treatment effects and the wide variety of estimation methods that can be applied to each framework. We consider why machine learning methods have been widely proposed for estimation and review the strengths and weaknesses of these approaches. We apply a range of these methods in re‐analyzing a landmark case study. In the application, we examine how several widely used methods may be subject to bias from model misspecification. We conclude with a set of recommendations for practice.

**Abbreviations**
NRS: non‐randomized studies
PS: propensity score
RCTs: randomized controlled trials

## 1 Introduction

Comparative effectiveness research (CER), provides important evidence for regulators, reimbursement agencies, clinical decision‐makers, patients, and the public about the relative effectiveness of alternative treatments, which encompasses alternative drug treatments, but also includes different prevention strategies, diagnostic testing, devices, forms of surgery, rehabilitative techniques, public health interventions, innovations in health care delivery, organization, and financing. The primary aim of CER is to quantify the causal effect of an intervention on outcomes [1]. For example, is care at a hospital that is certified to have high quality nursing superior to a hospital that does not? Answering such questions is challenging since causal inference requires assessing not just how things are, but how things *would have been*. To learn about effectiveness, one must also consider what *would have happened* under different circumstances (e.g., if those treated had taken control). Causal inference offers a framework for formulating these questions mathematically, exploring whether answers can be gleaned from data, and if so, determining how well and with what statistical methods. Research designs and statistical methods for causal inference form a key part of CER. While well‐conducted randomized controlled trials (RCTs) are the primary method to estimate causal effects while avoiding bias due to confounding, in many settings RCTs cannot be conducted for ethical or practical purposes. As such, evidence from non‐randomized studies (NRS) is a critical component in decision‐making, so it is vital that they are designed, analyzed, and interpreted appropriately [2].

One key challenge in any NRS, is that when subjects select into treatments, outcomes may reflect pretreatment differences between the treatment and control groups rather than treatment effects [3, 4]. Pretreatment differences between the comparison groups may be measurable and result in overt bias. A further concern is that there may be baseline differences in unmeasured characteristics, for example people's lifestyle or behavioral characteristics that lead to hidden bias in the estimates of comparative effectiveness. A common strategy in NRS is to assume there is no hidden bias and apply a statistical adjustment strategy to remove overt bias. Traditionally, regression models were the only method of statistical adjustment used for this purpose. That is, researchers regressed an outcome on a treatment indicator and adjusted for a set of baseline prognostic measures also known as control variables to account for pretreatment differences between the comparison groups in those covariates. However, over the last twenty years, there has been an explosion in the number and range of methods that researchers can use for statistical adjustment. For example, one common alternative to regression models is matching, and there are now many different matching methods that can be applied, including propensity score matching, genetic matching [5], optimal matching [6], full matching [7], mixed integer matching [8], cardinality matching [9], optimal matching with refined covariate balancing [10], coarsened exact matching [11, 12], and kernel matching [13, 14]. Moreover, there are large number of alternatives to matching. These alternatives include a variety of weighting estimators, outcome modeling via the parametric g‐formula, and doubly robust methods. In addition, a large number of methods based on machine learning (ML) have been proposed for statistical adjustment and the estimation of causal effects.

A critical question in a study of causal effects, is how to choose from amongst the multitude of methods available for statistical adjustment? In this tutorial, we review the logic behind the range of methods available for applications with point treatments. Readers interested in settings with time‐varying treatments should refer to Daniel et al. [15]. We begin by reviewing the concept of model misspecification due to incorrect functional form assumptions, and how it can lead to biased estimates of treatment effects. We discuss how the possibility of bias from model misspecification has motivated the growth in methods for statistical adjustment. Specifically, the overall trend in methodological development has been towards more flexible non‐ and semi‐ parametric forms of adjustment to reduce the need for a correct model specification. We conduct a simulation study that provides a clear rationale for considering more flexible methods of estimation. Next, we review the key choices for selecting a method of statistical adjustment. First, we outline the three different modeling approaches for treatment effects. Second, we review the large number of estimation methods that can be used to implement each approach. We also focus on why machine learning based methods have become so widely proposed. We explain both the theoretical and practical advantages and disadvantages of the options available to applied researchers. Finally, we re‐analyze a case study, to demonstrate how to implement these methods and highlight the strengths and weaknesses of these various approaches. In general, our review is conceptual and seeks to explain how different estimation methods encode different functional form assumptions. As such, we do not focus on how to implement all these methods in software. That is, our primary goal is to explain the key assumptions behind the various choices rather than focus on software specifics. Other work focuses more directly on software implementation [16]. However, to help analysts use these methods in their own research, we include a software appendix, which contains the codes used to generate the results in the application. In addition, a full set of replication materials are available online at https://github.com/ljk20/somanychoices. In the next section, we outline the details of our case study.

### 1.1 Application: Right Heart Catherization

We use data from a well‐known NRS that aimed to evaluate the comparative effectiveness of Right Heart Catherization (RHC) a monitoring device that is used in the management of critically ill patients [17]. In this study, the researchers included eligible patients admitted to Intensive Care Units (ICU) in the USA, and compared the effect of ‘RHC’ versus ‘control’ (no RHC) on all‐cause mortality at 6 months. The study included 5735 critically ill adult patients of whom 2184 had a RHC inserted (‘RHC’ group), and 3551 who did not have a RHC inserted and form the control group. For full details about the study readers are referred to [17]. Here, we outline the key features relevant for the subsequent analyses. The data contain a rich set of baseline covariates: sex, probability of 2‐month survival, coma score, an indicator for do not resuscitate status, the APACHE III acute physiology score, education, an index of daily activities 2 weeks prior to admission, Duke Activity Status Index, physiological measurements, ethnicity, income, insurance class, primary disease category, admission diagnosis, an indicator for cancer, PaO_2_/FiO_2_ ratio, creatinine, PaCO_2_, albumin, number of comorbid illnesses, temperature, respiratory rate, heart rate, and white blood cell count. The primary outcome is 6‐month mortality. Previous re‐analyses of this case study have all suggested that, given the richness of the baseline covariates, it was plausible to assume no unobserved confounding. However, this study exemplifies the major general concern in such settings, that it is also necessary to make assumptions about the functional relationships between each of the baseline covariates, treatment, and the outcome. In trying to address this concern, it is unclear, how the analyst should proceed in choosing from amongst groups of methods (e.g., outcome, treatment, or doubly robust models) or indeed from the estimators within the broad groups. In the next section we review the general concepts for all of the methods.

## 2 Review: NRS

First, we review the relevant concepts for NRS. We primarily focus on how bias can result from model misspecification in an NRS. Model misspecification bias is a key concept, since it motivates the wide ranging set of methods that serve as alternatives to regression models. First, we outline notation and causal estimands.

### 2.1 Notation and Estimands

In the RHC study, the patient population is indexed by i=1,…,n, and we denote a binary treatment using $Z_{i}$ where ($Z_{i}=1$ (RHC), $Z_{i}=0$ (control)). We use $Y_{i}$ for the binary mortality outcome. Next, we use the potential outcomes framework to describe causal quantities [4, 18]. Prior to treatment, each patient has two potential responses: $\left(Y_{i}(1),Y_{i}(0)\right)$. The outcomes that we actually observe are a function of potential outcomes and treatment assignment: $Y_{i}=Z_{i}Y_{i}(1)+(1-Z_{i})Y_{i}(0)$. We have a large number of pre‐treatment covariates for each patient, which we describe with $X_{i}$. For each patient, there is possibly an unobserved covariate $u_{i}$ that functions as a hidden confounder.

In this framework, we first define the causal effect—that is, the estimand—of interest. Estimands are defined as contrasts of potential outcomes. Two common estimands targeted in a NRS are the average treatment effect (ATE) and the average treatment effect on the treated (ATT). The formal definition of the ATE is

(1) $$ATE=𝔼\left[Y_{i}(1)-Y_{i}(0)\right]$$

which is the average difference in the pair of potential outcomes averaged over the entire population of interest. In the context of the RHC application, the ATE measures the average difference in mortality when all patients in the study population are assigned to RHC versus when all patients are assigned to control. Often, the average treatment effect is defined for the subpopulation exposed to the treatment or the ATT:

(2) $$ATT=𝔼\left[Y_{i}(1)-Y_{i}(0)|Z_{i}=1\right]$$

The ATT is the average difference in potential outcomes among those individuals in the population that were actually exposed to the treatment. These estimands answer different scientific questions, so investigators must select which to target based on substantive judgements. In the RHC application, we focus on the ATT, since as Connors et al. [17] highlight there was strong clinical interest in whether PAC insertion should be stopped for the subpopulation who had the device. In other settings the ATE may be the estimand of interest [19]. See Ben‐Michael and Keele for further discussion about the choice of estimand [20].

### 2.2 Assumptions

Next, we outline the standard set of assumptions that are invoked to identify the ATT in a NRS. First, we assume the stable unit treatment value assumption (SUTVA) holds [21]. SUTVA is comprised of two components: (1) the treatment levels of Z (1 and 0) adequately represent all versions of the treatment, often referred to as the consistency assumption in the epidemiology literature [22], and (2) a subject's outcomes are not affected by other subjects' exposures. Next, we must assume that treatment assignment is independent of the potential outcomes conditional on the observed covariates. This assumption has a number of different names, which include “conditional ignorability,” “conditional exchangeability,” “no unobserved confounding,” and “no omitted variables.” In the language of causal diagrams, researchers must identify all backdoor paths between baseline covariates, treatment and the outcome [23, 24]. Formally, we assume that treatment assignment only depends on observed covariates:

$$Pr(Z_{i}=1|Y_{i}(1),Y_{i}(0),X_{i},u_{i})=Pr(Z_{i}=1|X_{i})$$

If this assumption is implausible, other study designs might be more reasonable [25, 26]. Next, we assume the probability of treatment is strictly greater than zero and less than one over the support of $X_{i}$:

$$0<Pr(Z_{i}|X_{i})<1$$

This assumption is often referred to as overlap, common support, or positivity. Note that when overlap between the treated and control populations is limited, the ATT may be identifiable when the ATE is not. For some data configurations, overlap may be so limited that even the ATT may not be identifiable. When this occurs, one strategy is to use an alternative estimand that only targets the subset of treated units that overlap with the control units [27, 28, 29]. One such estimand is the average treatment effect for the overlap population (ATO) [29]. Under the ATO, the estimand is focused on the marginal population that might or might not receive the treatment of interest rather than a known, a priori well‐defined population such as the treated group.

This set of assumptions becomes implausible when units are selected into treatments based on prognostic factors that indicate who would benefit more from a specific treatment, but not all those prognostic factors are recorded. While we assume that there are no unobserved differences in such baseline prognostic measures between the treated and control groups, the broad aim of the statistical methods that we consider is to adjust, match or reweight these groups so that they are similar according to observed baseline measures. For example, in the RHC application, there are clear differences in observed baseline characteristics between the treatment and control groups. Table 1 contains balance statistics for the set of covariates with the largest imbalances. We observe clear differences between the treated and control groups. For example, prior to PAC insertion patients in the RHC group are more likely to have a cardiovascular diagnosis or multiple organ failure with sepsis than those in the control group. One rule of thumb is that standardized differences should be less than 0.20 and preferably 0.10 [30]. Clearly according to this rule of thumb, many of the baseline differences are quite large. To estimate the treatment effect for RHC, we must remove such differences via statistical adjustment. Next, we review the concept of model misspecification and demonstrate how it can be a key threat to valid causal inferences.

**TABLE 1:** Balance table for baseline covariates in the RHC versus control groups for the Connors et al. example: RHC: Selected covariates with largest imbalances.

	Mean RHC	Mean control	Std dif
Respiratory diagnosis (0/1)	0.29	0.42	−0.27
Cardiovascular diagnosis (0/1)	0.42	0.28	0.29
Neurological diagnosis (0/1)	0.05	0.16	−0.35
APACHE III score	60.74	50.93	0.50
Weight (kg)	72.36	65.04	0.26
Mean blood pressure	68.20	84.87	−0.46
PaO_2_/FiO_2_ ratio	192.43	240.63	−0.43
PaCO_2_	36.79	39.95	−0.25
Hematocrit	30.51	32.70	−0.27
Creatinine	2.47	1.92	0.27
Acute renal failure (0/1)	0.03	0.11	−0.34
Multiple organ failure w/ sepsis (0/1)	0.32	0.15	0.41

## 3 Model Misspecification

When investigators estimate treatment effects, there are two possible sources of bias. We use the following equation to describe these two possible sources of bias in the estimation of causal effects:

$$\begin{array}{ll} & \text{Estimator}-\text{True causal effect}\\ & \;=\underset{\text{Due to design}}{\underbrace{\text{Hidden bias}}}+\underset{\text{Due to modeling}}{\underbrace{\text{Misspecification bias}}}\\ & \;+\underset{\text{Due to finite sample}}{\underbrace{\text{Statistical noise}}} \end{array}$$

In a NRS, we assume that under the conditional exchangeability assumption, hidden bias is not present. Here, we use hidden bias to refer to bias from unobserved confounders and measurement error. This bias is hidden, since we cannot know its true magnitude. If those assumptions are implausible other study designs might be more reasonable [25, 26]. Here, we focus on a different form on bias, misspecification bias that is a consequence of using an incorrect model for statistical adjustment. Next, we unpack what it means to use an incorrect model for adjustment. Estimation of treatment effects consists of specifying a model for the conditional mean function of the outcome. That is, the treatment effect is the difference in two conditional expectations for the outcome:

$$E[Y_{i}|Z_{i}=1]-E[Y_{i}|Z_{i}=0]$$

This estimator for this difference in conditional expectations can be written as the following restriction on the conditional mean function:

$$E[Y_{i}|Z_{i}]=\lambda_{0}+\lambda_{1}Z_{i}$$

This restriction on the conditional mean function is a model, since it places an *a priori* restriction on the joint distribution of $Y_{i}$ and $Z_{i}$, and it is referred to as the functional form of the model. Functional form restrictions are often referred to as parametric models, since the model depends on the terms $\lambda_{0}$ and $\lambda_{1}$, which are the parameters in this model. This model is saturated, since the number of parameters in the model is equal to the number of unknown conditional means. That is, the two means to be estimated are $E[Y_{i}|Z_{i}=1]$ and $E[Y_{i}|Z_{i}=0]$, and there are two parameters in the model. In an NRS, the model for the conditional mean function is often written as

$$E[Y_{i}|Z_{i},X_{i}]=\lambda_{0}+\lambda_{1}Z_{i}+\lambda_{2}X_{i}$$

where $X_{i}$ represents an observed confounder. The functional form for this conditional mean model is linear, since we assume that the changes in the conditional mean of $Y_{i}$ as a function of $Z_{i}$ and $X_{i}$ are best described by a straight line. The functional form of the model encodes a set of assumptions by the investigator about how the conditional mean varies with the treatment and confounders. Note that if $X_{i}$ is an indicator variable, and if we include an interaction term between $Z_{i}$ and $X_{i}$, the model remains saturated and does not impose any additional restrictions on the conditional mean function. If $X_{i}$ consists of a continuous covariate, $X_{i}$, the model is no longer saturated and encodes parametric restrictions on the conditional mean function. To understand the assumption encoded into this functional form, we write the model in a more general way:

$$E[Y_{i}|Z_{i},X_{i}]=\lambda_{0}+\lambda_{1}Z_{i}+\lambda_{2}g(X_{i})$$

In this more general specification, g() represents possible functions for $X_{i}$. In the first model, g() is assumed to be the identity function, but many other functions are possible. That is, g() could be quadratic or cubic if $Y_{i}$ varies with $X_{i}$ in a nonlinear fashion. The choice of g() imposes a functional form restriction, since it limits how the conditional mean of $Y_{i}$ varies with $X_{i}$. Model misspecification arises when the investigator selects a model with an incorrect set of restrictions, for example, the relationship between $Y_{i}$ and $X_{i}$ is assumed to be linear when it is nonlinear. Another possible form of model misspecification is when the treatment effect varies with $X_{i}$. In this case, model misspecification occurs when a relevant interaction between $Z_{i}$ and $X_{i}$ is not included in the model. In sum, misspecification bias refers to bias from misspecifying the functional form of the statistical model.

Model misspecification can be viewed as under‐specification. For example, if g() is quadratic and only a linear term is included, the model is under‐specified, since an additional parameter hasn't been included for the second moment of $X_{i}$. See Lenis, Ackerman, and Stuart [31] for a method to measure the amount of model misspecification. However, one can also over‐specify the model by including irrelevant parameters. For example, by including a squared term when the relationship between the covariate and the outcome is linear. The additional parameter for the squared term, is irrelevant and represents an over‐specification. The consequence of over‐specification is to inflate the variance which will make the 95% confidence intervals wider.

### 3.1 A Simulated Example

Next, we conduct a simulation study to demonstrate how model misspecification can bias estimates of treatment effects. The data‐generating process we use is based on one presented in Goff [32]. In our simulation, the model for the conditional mean of the outcome has the following form:

$$Y=Z_{i}+g(X_{i})+\epsilon$$

where $Z_{i}$ is the treatment variable, $X_{i}$ is an observed confounder, g() is the functional form for $X_{i}$, and ϵ is a normally distributed error term. We set the ATE to 1, and we specify $g()=X_{i}^{2}$, so that X has a nonlinear association with Y. Consistent with what we would expect in a NRS, $X_{i}$ is a confounder that is associated with both $Y_{i}$ and $Z_{i}$. We generated $Z_{i}$ and $X_{i}$ as draws from a multivariate normal distribution with variances 1.5 and 6 respectively. We set the correlation between Z and $X_{i}$ to 0.50. Therefore, $X_{i}$ must be included in the model to consistently estimate the ATE. We set the sample size and the number of simulation replications to 1,000. In the simulation, we included three models with different specifications. In the first, we omit $X_{i}$ entirely. We expect the treatment effect $Z_{i}$ to be biased for this specification, since the key confounder is omitted. In the second specification, we introduce model misspecification by only including $X_{i}$ in the statistical model. Here, we have incorrectly specified the functional form of the model by omitting the quadratic functional form for $X_{i}$. A quadratic term is often a plausible nonlinear functional form, since it captures a rapidly changing effect that tapers off after some threshold is reached. In the final specification, we include $X_{i}^{2}$ in the model so that the functional form of the model is now correctly specified.

**FIGURE 1:**
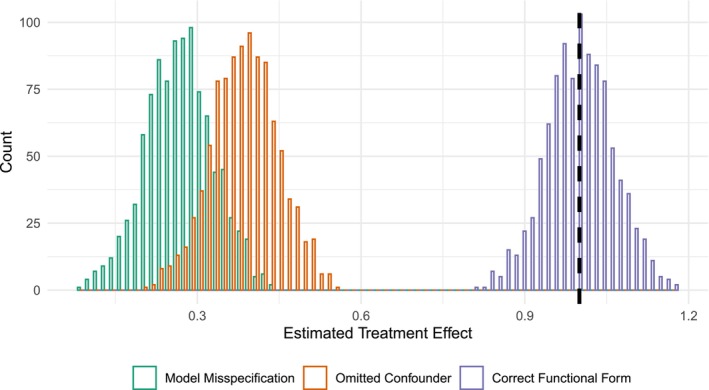
Simulation results for different specification scenarios. Dashed line represents true treatment effect.

Figure 1 contains the results from the simulation. For each model, we plot the distribution of estimated treatment effects. First, we observe that when the key confounder is included in the model *and* the correct functional form is used, we recover the true treatment effect as the distribution of estimates is centered at the true effect. Next, if we omit the key confounder from the model, the estimated treatment effect is biased as it is uniformly too small, and the distribution of estimated effects is bound away from the true treatment effect. Finally, when we mis‐specify the model, the treatment effect is also biased. In fact, the average bias under model misspecification is somewhat larger than the bias from omitted confounder. Goff [32] shows analytically that the bias depends directly on the correlation between X and Z and the magnitude of the variances for X and Z. The bias also depends on g(). Hence, under certain conditions, bias from model misspecification can rival if not exceed the bias from omitted confounders. Avoiding bias from model misspecification is therefore a critical step in the estimation of treatment effects. As we outline below, the possibility of this type of misspecification bias has driven a number of innovations for the modeling of treatment effects.

## 4 Statistical Modelling of Treatment Effects

Thus far, we have outlined how model misspecification may be a significant source of bias when estimating treatment effects. Next, we review the key choices that are necessary for estimating treatment effects. First, we discuss the choice for the model of conditional expectations. Next, we focus on the choice of statistical estimators that can be selected within each framework of conditional expectations. Here, we focus on how non‐ and semi‐parametric estimation methods can reduce bias from model misspecification.

The first step for an analyst is deciding on which conditional expectation should be modeled. The traditional approach to statistical adjustment is to model the conditional expectation of the outcome, $Y_{i}$, given $Z_{i}$ and $X_{i}$. More formally, the model for treatment effects is based on the following conditional expectation: $y(x)=E[Y_{i}|Z_{i},X_{i}]$. Alternatively, one can model the conditional expectation of treatment, often called the propensity score (PS); that is, $Z_{i}$ given $X_{i}$: $e(x)=E[Z_{i}|X_{i}]$. Finally, one can model the conditional expectation for both the outcome and treatment. Under this approach, separate models are fitted for both y(x) and e(x), and the results are combined to estimate the treatment effect. Frequently, this approach is called “doubly robust,” since treatment effects are consistently estimated when either model for the conditional expectations is correctly specified [33]. As such, the investigator's first key choice is which conditional expectation to estimate.

The analyst also needs to make two additional choices related to model specification. Using the notation from above, for each variable in $X_{i}$, the investigator needs to select the functional form as represented by g(). For multi‐valued variables, selection of g() is focused on whether to include additional terms to allow for nonlinearity. For all types of variables, specification of g() also includes whether to include interactions between the variables in $X_{i}$. The second model specification choice is with respect to effect heterogeneity. This refers to whether or not we can assume that treatment effect is constant with respect to $X_{i}$. If we assume the treatment effect is non‐constant, we can take a conditional or marginal approach. Under the conditional approach, one estimates treatment effects at specific values of the variables in $X_{i}$. Under the marginal approach, one averages over the variation in the *Z*_*i*_‐*X*_*i*_ relationship. As we outline below, different methods of estimation entail different choices with respect to model specification.

### 4.1 Estimation Methods

Critically, all three approaches require the use of an estimation method for one or more of the conditional expectations of interest, but are agnostic as to the specific method of estimation. Before reviewing specific types of estimation methods, we introduce a distinction between fully parametric and semiparametric methods for estimating treatment effects. The difference between fully parametric and semiparametric methods is that semiparametric methods do not fully specify the relationship between $X_{i}$ and $Y_{i}$. For example, in the following model:

$$E[Y_{i}|Z_{i},X_{i}]=\lambda_{0}+\lambda_{1}Z_{i}+\lambda_{2}X_{i}$$

the parameters $\lambda_{0}$ and $\lambda_{2}$ can be treated as nuisance parameters, since they are incidental and do not describe the treatment effect of $Z_{i}$ on $Y_{i}$. Under a fully parametric approach, the relationship between $X_{i}$ and $Y_{i}$ is specified in the statistical model, and the parameters $\lambda_{0}$ and $\lambda_{2}$ are estimated. The common feature of semiparametric approaches, is that these nuisance parameters are not estimated. By not fully specifying the model for the control variables, these approaches may reduce the likelihood of bias from model misspecification for $X_{i}$. We first focus on the most widely used parametric and semiparametric methods and highlight the key differences between various forms of semiparametric methods.

The traditional approach for estimating treatment effects is based on regression models for the outcome. We use the term regression model to encompass linear regression via least squares but also generalized linear models such as logistic or Poisson regression. Critically, these regression approaches are the most restrictive in terms of the functional form, since they are all fully parametric. That is, with regression models, the analyst must fully specify the relationship between $X_{i}$ and $Y_{i}$. As the number of variables in $X_{i}$ grows the likelihood of model misspecification typically increases. However, if all the variables in $X_{i}$ can be expressed as a series of dummy variables, then regression models are closer to being saturated, and the risk of model misspecification may be lower. We should note that for regression models the treatment effect is assumed to be constant, unless the full range of *Z*_*i*_‐*X*_*i*_ are include in the model.

There has been a huge expansion in the number of alternatives to parametric regression—all of which are semiparametric methods. We would argue that there are five main classes of alternative estimation methods: standardization, matching, weighting, doubly robust methods, and machine learning methods. Matching and weighting are the most widely used alternatives to parametric regression models. It is worth noting that the lines between these three forms of estimation are porous and often overlap. For example, one can use machine learning methods to estimate treatment effects via an outcome model or instead use them to estimate the propensity score and then implement either matching or weighting methods. In addition, doubly robust methods can be based on matching, weighting, or machine learning. However, all of these methods share a commonality in that they all treat control covariates coefficients as nuisance parameters.

#### 4.1.1 Standardization

Standardization via the parametric g‐formula is a semiparametric method for adjustment using an outcome model that treats the parameters for control variables as nuisance parameters [34]. Hernán and Robins [35], ch. 13 provide a complete and accessible introduction to the parametric g‐formula. Here, we include a brief outline. Standardization via the parametric g‐formula operates by fitting separate outcome models by treatment status and then marginalizing over the predicted outcomes for each of these models. The difference in these marginalized predictions is the treatment effect estimate. Inference for the parametric g‐formula proceeds via the bootstrap. Both the parametric g‐formula and standard regression modeling can allow for non‐constant treatment effects according to the levels of the covariates. However, one distinction between these approaches is whether effect heterogeneity is treated as marginal (parametric g‐formula) or conditional (standard regression modeling).

#### 4.1.2 Matching Methods

Matching may be the earliest proposed alternative to traditional regression methods. Matching methods are designed to model e(x), and the estimated parameters for baseline covariates are treated as nuisance parameters. Early versions of matching were unable to control for large numbers of covariates [36, 37], but matching became a more viable technique with the application of multivariate distance metrics [6]. In general, matching requires the calculation of a distance matrix that contains measures of covariate similarity between each treated unit and all potential control units. Propensity score distances and the Mahalanobis distance are frequently used to measure similarity between units. Early matching methods created pairs by searching over these distances. Later, optimization methods were used to find treated to control assignments that minimize the total distances between two groups [6]. While pair matching is the most common, matched strata can take many forms depending on the study design [7, 38, 39, 40]. Recently, many different matching methods have been proposed, from those that more general [5, 8, 12, 40] to others that focus on specific problems in statistical adjustment [9, 10]. There are a variety of ways that treatment effects can be estimated after matching is complete, and most assume the treatment effect is constant.

#### 4.1.3 Weighting Methods

The next major class of methods is ‘weighting estimators’ which are commonly based on the traditional inverse probability weighting (IPW) estimator [27, 41, 42, 43, 44, 45]. The IPW estimator, like matching, is a semiparametric method based on e(x) and also treats the parameters for $X_{i}$ as nuisance parameters. The IPW estimator is based on weighting treated and control units by the estimated propensity score. Weighting units by the estimated propensity score, in expectation, balances the distribution of $X_{i}$ across the treated and control groups [42]. Critically, while the balancing property for the true propensity score weights holds in expectation, it may not hold in any particular data set. Moreover, if the model for the estimated propensity score is misspecified then by definition IPW will not achieve the required covariate balance. A newer class of weighting methods solve a convex optimization problem to find a set of *balancing weights* [46, 47]. Balancing weights are designed to directly target covariate balance in the estimation process. Theoretical work has shown that balancing weights are implicitly estimates of the inverse propensity score, fit via a loss function that guarantees covariate balance [48, 49, 50, 51]. One common way to implement the IPW estimator is via marginal structural models [52]. Here, the IP weights are used in a weighted outcome model in which the outcome is regressed on the treatment indicator. Under this approach, the treatment effect is assumed to be constant. See Zubizaretta et al. [53] for a detailed overview on both matching and weighting methods.

#### 4.1.4 Doubly Robust Methods

DR estimators were first developed as extensions of the IPW estimator, and were referred to as the “augmented” inverse propensity score weighted (AIPW) estimator [33]. The AIPW estimator is based on two steps. First, IP weights are estimated. Next, two outcome models are fitted: one for the outcome under treatment and one for the outcome under control. These two outcomes are weighted by the propensity score to produce an estimate of the treatment effect. Note that the AIPW estimator provides a marginal estimate that does not assume the treatment effect is constant. However, one can implement DR estimators in alternative ways. For example, another version of the DR estimator is based on estimating an outcome model using a matched data set. This outcome model includes confounders in the specification to further reduce bias not eliminated by matching [54]. Alternatively, when using the IPW estimator, one can include additional covariates in the marginal structural model for additional bias correction. See Hernán and Robins [35] for an approachable review of how DR methods work.

Next, we review how doubly‐robust methods can reduce model misspecification. For this exercise, we outline the following set of equations for e(x) and y(x) to structure the discussion:

$$e(x)=\tau_{1}X_{1}+\tau_{2}X_{2}$$

$$y(x)=\beta Z_{i}+\lambda_{1}X_{2}+\lambda_{2}X_{3}$$

Here, treatment assignment depends on $X_{1}$ and $X_{2}$, and the outcome on $X_{2}$ and $X_{3}$. Next, we assume that $X_{1}$, $X_{2}$, and $X_{3}$ do not have any common causes. What advantages do DR methods offer in this context? DR methods offer two advantages in this context. First is with respect to variable specification. That is, if the analyst were to decide to model e(x) but omit $X_{2}$ from this model, the treatment effect estimate would be biased. If the analyst were to model y(x) but omit $X_{2}$ from the model, the estimate of the treatment effect would be biased. Under the DR framework, the analyst would be able to consistently estimate the treatment effect as long as $X_{2}$ is included in either y(x) or e(x). Critically, DR methods also offer protection against model misspecification. As we outlined above, analysts needs to specify the correct functional form for either e(x), y(x), or both. If the analysts decided to take an outcome focused approach, he or she would need to correctly specify g() in y(x). For the PS approach, the analyst would need to correctly specify g() in e(x). However, for the DR approach, g() needs to be correct in only one of the two models. That is, as long as the functional form is correct in one of the two models, the treatment effect will be consistently estimated. The obvious advantage of the DR approach is that it provides consistent estimates of the treatment effect while allowing for some form of model misspecification. However, DR methods are not a panacea, in that g() could be wrong in both models. In this case, it is hard to predict which approach will be superior. The relative performance will strongly depend on the data generating process and either a y(x) or e(x) approach may very well outperform DR methods [55]. As such, there are no guarantees that a DR approach is automatically superior.

One alternative is to implement the outcome and propensity score based approaches separately. Large differences between these two estimates will alert the analyst to the presence of serious model misspecification in one of the approaches. Alternatively, Mercatanti and Li [56] suggest using DR estimates as a benchmark. If the DR estimates are close to those based on e(x) but far from those based on y(x), then the outcome model is likely misspecified. If the DR estimates are close to those based on y(x) but far from those based on e(x), then the propensity score model is likely misspecified. If the DR estimates are far from both estimates of y(x) and e(x), and the estimates from y(x) and e(x) also differ from each other, it will be difficult to say which modeling approach is correct [55]. Ideally, estimates are consistent across all three approaches. In general, we recommend that analysts always take this more agnostic approach instead of selecting a single approach. What concrete recommendations might we offer in terms of reporting statistical results? If all three methods agree, it is still important that the full set of results are available for inspection albeit with some results as supporting material. When results disagree, however, readers should include all three sets of results in the main text. Investigators should also attempt to offer explanations as to the likely reasons for the differences across the sets of methods in the treatment effect estimates. In the application section, we demonstrate how differences across the approaches can be reconciled.

#### 4.1.5 Machine Learning Methods

While nuisance parameter approaches reduce the likelihood of model misspecification relative to fully parametric models, they are not a panacea for model misspecification. As we noted above, however, nuisance parameter approaches still require important model specification choices by the analysis. That is, most nuisance parameters approaches require the analyst to specify g() and decide whether to allow effect heterogeneity. For example, with matching, the analyst must decide whether to match on additional terms to account for nonlinearities in continuous covariates. In addition, the analyst must identify any relevant interactions between the covariates and include those interactions in the distance matrix. Alternatively, many IPW estimators are often implemented with parametric logistic regression models. The logistic regression model used for the propensity score must be correctly specified to avoid model misspecification. Specifically, this logistic regression model must include terms for possible nonlinearities and interactions between covariates. In addition, many common matching and weighting methods assume the treatment effect is constant with respect to $X_{i}$. DR methods have the same model specification issues for each of the underlying models. To prevent model misspecification, Imbens and Rubin [57], ch. 13 outline an iterative process for selecting relevant interactions and nonlinear terms. However, when there are a large number of possible confounders, this process becomes very time consuming and cumbersome. To further reduce the likelihood of model misspecification, researchers have proposed using nonparametric or semiparametric estimators widely referred to as “machine‐learning” (ML) methods. Next, we review how ML methods have been employed to further reduce the likelihood of model misspecification.

In general, ML methods are nonparametric estimation methods that use richly parameterized models to fit conditional expectations. Traditionally, ML methods have employed for statistical prediction problems, but they are easily adapted to nuisance parameter approaches to treatment effect estimation. In one early example using ML methods, McCaffrey, Ridgeway, and Morral [58] used gradient boosting machines (GBM) to flexibly model the PS. Here, an ML method is used to estimate the PS, which is then used with a standard IPW estimator. Why is this advantageous? As we noted above, for an IPW estimator, we must fit a model for e(x), and this model can be misspecified especially in terms of selecting interactions and nonlinear terms. Tree based methods such as random forests and GBM are designed to automatically include relevant interactions for variables included in the model. As such, using a GBM to estimate the PS model, can reduce model misspecification, since the analyst is not required to identify relevant interactions or nonlinearities. Another prominent example of using ML for treatment effect estimation is Hill, Weiss, and Zhai [59] who proposes using Bayesian additive regression trees (BART) to flexibly model y(x). This proposal focuses on modeling the outcome, but uses a flexible ML method instead of a more restrictive parametric model. Finally, DR estimators have been estimated with various forms of ML methods [60, 61].

One way to conceptualize how ML methods reduce bias from model misspecification is to view them as using many parameters to describe the conditional expectation of interest. For example, let's assume that in a model for the outcome, we suspect quadratic nonlinearity for a covariate. We would then specify a model with three parameters instead of two for a linear model. If the model for the outcome is actually linear, adding an additional parameter for the quadratic term will not add any bias. However, it will increase the estimated variance slightly—this model will have somewhat wider confidence intervals. In general, statistical models can be made more flexible by estimating additional parameters, but this comes at a cost of higher variance. More generally, ML methods can be viewed as a set of statistical methods that estimate many parameters to flexibly model conditional expectations. More specifically, ML methods can use the data to specify g() in y(x), e(x), or both. That is, ML methods can specify nonlinearity or interaction in g(). This flexibility reduces or may even eliminate bias from model misspecification, and reduces the need for analysts to make ad hoc choices for g().

The advantages of ML methods are obvious in terms of reducing the risk of bias from model misspecification. Unfortunately, using ML methods for estimating treatment effect raises additional complexities. In general, ML methods rely on hyperparameters to control the tradeoff between complexity and variance. That is, letting an ML method select a highly complex functional form will tend to reduce bias, but may increase the variance drastically. Less complex ML fits will tend to allow some bias but reduce the variance of the estimate. The hyperparameter(s) control this tradeoff. For example, the lasso uses a hyperparameter, typically referred to as λ, to control the bias variance tradeoff. If the analyst specifies large values of λ this will permit increased bias but decrease the variance, while specifying smaller values of λ allows for more complex models with additional parameters and so decreases the bias while increasing the variance. One common data driven hyperparameter selection method uses a mean‐squared error (MSE) criterion. That is, the analyst can select a hyperparameter value so as to minimize the squared bias added to the variance. Let's say we use a MSE criterion to select the hyperparamter value. This implies that the fit contains some amount of bias based on the MSE criterion. Any bias included in the fit due to this hyperparameter value is often referred to as smoothing bias [62, 63]. Hence the resultant ML estimates of the treatment effect will incorporate this smoothing bias, which has the potential to be quite large [62, 63]. The added flexibility of ML methods also comes at a cost in terms of inferential properties. In general, for ML methods it is difficult to obtain valid inferences. That is, for many ML methods the associated statistical tests and confidence intervals may not be valid. In addition, ML methods may be very inefficient relative to less flexible methods [62, 64, 65, 66]. As such, ML methods are no panacea. While ML methods provide flexible fits for y(x) and e(x), treatment effect estimates remain biased or have poor inferential properties.

However, a new approach has been developed that uses flexible nonparametric ML estimation methods, but reduces the threat of smoothing bias and allows for valid statistical inferences that are optimally efficient. This framework is built from a combination of semiparametric theory, doubly robust methods, and machine learning methods [62, 64, 67, 68]. We refer to this framework as the doubly robust machine learning (DRML) framework. The framework starts by constructing bias‐corrected, doubly‐robust estimator using influence functions from semiparametric theory. DR estimators based on influence functions allow for estimation via ML methods that account for smoothing bias, under mild conditions. This estimation framework is then combined with sample‐splitting or cross‐fitting to obtain doubly robust estimates based on ML methods that have known statistical properties [65, 66, 69]. One prominent example of a DRML method is targeted maximum likelihood estimation (TMLE) [70, 71]. Here, we provide a brief overview of TMLE to illustrate one way that DRML estimation methods can be implemented. The first step in TMLE is based on fitting a model for the outcome. Critically, this outcome model can be fit via an ML method such as random forests. Alternatively, the analyst can take a weighted average of fits from an ensemble of ML methods, one well‐known ensemble is the Superlearner [72]. In the second step, the analyst fits a propensity score model typically via an ML method. In the third step the analyst estimates what is known as the fluctuation parameter. This step uses information in the fitted propensity score to optimize the bias‐variance tradeoff in the estimand of choice. The fluctuation parameter is used to update the information in step to provide a final estimate of the treatment effect. Finally, inferential quantities are computed. See Gruber et al. for an applied guide to using TMLE [73].

In sum, the DRML framework can allow for the estimation of treatment effects based on flexible machine learning methods to reduce bias, and some of these approaches have valid inferential properties. Critically, this framework does not depend on the type of ML method used. In sum, the DRML framework offers some key advantages. It allows the analysts to use flexible ML methods to reduce model misspecification, but avoid bias due to smoothing bias from those ML methods.

### 4.2 Summary

To summarize, researchers must make two broad choices when estimating treatment effects. The first choice is selecting among the two conditional expectations. The second choice, which is more complex, is selecting among the estimation methods that can be employed. For any of the three sets of conditional expectations, one can then use anything from fully parametric models to highly flexible ML methods. The general conundrum is that parametric methods tend to be more familiar to applied investigators and easier to use in statistical software. If a parametric model has the correct functional form for the conditional expectation of interest, all these methods will produce consistent estimates of the treatment effect. However, if the functional form is more complex, more flexible methods can eliminate bias from model misspecification. While flexible ML methods may introduce bias from smoothing and their inferential properties may be poor, DRML methods can be used to allow for flexible fits that reduce smoothing bias and preserve inference.

Given the wide range of choices, the natural question for applied investigators is: what set of choices is best? Given theoretical results from the literature, DRML methods appear to be the best choice. That is, while ML methods are attractive in terms of flexibility, there are few guarantees in terms of inference outside of a DRML framework. As such, DRML methods would appear to be the logical choice. However, DRML method tend to be more complex in terms of implementation and can require lengthy computing times, which begs the question of whether less complex methods might be adequate.

We might ask whether there is any empirical evidence that sheds light on which method should be the first choice of applied analysts? While there is some evidence, that evidence tends to be mixed. Dorie et al. [74] conducted a contest where participants selected a variety of methods to fit to simulated data. In the contest, ML based methods were clearly superior. Notably, DRML methods did not appear to outperform other ML methods. Next, Keele and Small [75] compared a variety of ML based methods, including DRML, to matching methods and found little difference across five different empirical applications. Finally, Keele, OÃćâĆňâĎćNeill, and Grieve [76] applied a wide variety of statistical adjustment methods seeking to recover an experimental benchmark. In this study, DRML methods, again, did not tend to outperform more standard methods—including regression models. Next, we present the empirical example to identify insights from applying examples of the alternative approaches to address a clear causal question. It is worth remembering, however, that an absence of model misspecification in one application is not an argument against DRML methods. In those settings, DRML methods should produce the same results. In some other application, parametric methods may be biased, while DRML method are consistent.

## 5 Application

We now apply these approaches to an empirical application. As we outlined above, we use a well‐known data set on the effectiveness of RHC for the management of critically ill patients. An early study applied propensity score matching and found that compared to ‘usual care’ insertion of a RHC in critical care was associated with higher mortality rates [17]. A later investigation used more advanced matching methods to find the same empirical pattern [77]. However, a randomized controlled trial (RCT) found that RHC did not increase the risk of death [78]. The RCT reported that the proportion of people who died prior to hospital discharge was 68.4% for patients assigned to the RHC group, 65.7% for those assigned to the control (usual care) group, with an (unadjusted) estimated ATE of ‘RHC’ versus ‘control’ on mortality of 2.7% (95% CI‐3.1% to 8.5%) [79]. Note that in appropriately conducted RCTs the ATE and the ATT are the same, and also that while the outcome measures are different between the RCT (in hospital mortality) and the NRS (6‐month mortality), they are highly correlated. We also note that many of the baseline covariates are multi‐valued, which may increase the risk of bias from model misspecification, since parametric models will be unable to use saturated specifications.

We evaluate different methods of statistical adjustment by attempting to recover the RCT estimate using the NRS data. That is, we apply a range of statistical adjustment methods to the RHC data, and compare these estimates of comparative effectiveness to those from the RCT. Here, following on from the RCT result, we anticipate that the true causal effect is that compared to “control” or “usual care”, insertion of the RHC does not increase the risk of mortality to an extent that is significantly significant. This design depends on a number of key assumptions. First, we must assume that unobserved confounding is not present. Second, we have to assume that there are no key effect modifiers that differ between the RCT and NRS study populations. See Dahabreh, Robins, and Hernán [80] for a complete discussion of the key assumptions needed for benchmarking observational study results to an RCT. These assumptions become particularly relevant if we find that we cannot recover the RCT benchmark. For each method, we note the extent to which the observational study estimates agree with those from RCT, but do not conduct formal tests of equivalence.

Next, we outline the methods of statistical adjustment that we applied. We did not attempt to include a complete set of statistical adjustment methods. Instead, we selected methods based on key properties. Moreover, we group the methods in terms of overall flexibility. In the first set of statistical adjustment methods, we include standard regression models, standardization via the parametric g‐formula, and a IPW estimator, where we estimate the propensity score using a logistic regression model. These three methods represent methods of statistical adjustment that rely on strong function form assumptions and thus are at the most risk for bias from model misspecification. For these methods, we did not attempt to expand the specification with nonlinear terms or interactions.

The next two set of methods are more advanced forms of matching and balancing weights. For matching, we use a form of optimal matching with refined covariate (RC) balancing [10]. RC balancing is a matching method designed to use fine or near‐fine balance constraints [81, 82] to balance the joint distribution of many nominal covariates [10]. That is, it seeks to balance the marginal distributions of a large set of nested nominal covariates. It can also incorporate additional constraints via a caliper on the propensity score. We used RC balancing for several reasons. First, it allows analysts to prioritize which covariates have the smallest imbalances after matching. Next, RC balancing matches directly on covariate distances, and it provides a principled way to trim the sample by removing those observations that contribute most to imbalance. As such, RC balancing contains all the features of recent advances in matching. We should note that if RC balancing drops treated observations to improve balance this will change the estimand to a more local version of the ATT, and analysts should make this change to the estimand explicit.

For weighting, we use the balancer library in R [83]. This method of weighting directly targets covariate imbalance measured as the $L^{2}$ norm of the weighted difference in means of the covariates, but also includes an $L^{2}$ regularization term on the sum of the squared weights, which serves as a proxy for the variance of the weighting estimator. These weights include a hyperparameter that controls the bias‐variance tradeoff which is set by the user. Application of this weighting method are available in these publications [20, 84, 85]. As such, this weighting method directly targets balance. For both of these methods, we estimated treatment effects with and without outcome adjustment. That is, both matching and weighting are focused on the conditional expectation of treatment. For both, we can estimate an outcome model that includes covariates for additional bias reduction [57, 86]. These two adjustment methods do not impose the strong function form assumptions as the first two set of methods, but are not highly flexible like those based on ML methods. Again, we did not attempt to expand the specification of these two methods.

Next, we include two different DRML methods. The first DRML method we use is generalized random forests (GRF) [87, 88]. This DRML method adapted random forests, which are widely used for statistical prediction problems, to the estimation of treatment effects. Next, we use a DRML method based on a Super Learner (SL) combined with targeted maximum likelihood estimation (TMLE) [60, 71, 89]. For this DRML method, the analysis selects among a set of ML methods—learners—that will all be used as methods of statistical adjustment. For example, one might select GLMs, generalized additive models (GAMs), and random forests. The set of learners selected by the investigator are used to make out‐of‐sample predictions through cross‐validation. The predictions from each learner are combined according to weights that minimize the squared‐error loss from predictions to observations. These weights are then used to combine the fitted values from each learner when fit to the complete data. Then TMLE is applied to produce an estimate of the ATE or ATT. In the RHC application, we used the following set of learners: (1) GLMs, (2) GAMs, (3) random forests, (4) lasso, and (5) GBMs.

### 5.1 Results

Table 2 contains the results from the first set of methods: regression, standardization, and model‐based IP weighting. Interestingly, despite the fact that two of the methods are modeling the outcome and the other method is modeling the treatment, the results are nearly identical. All the methods estimate that RHC increases the risk of death by just over 6%, with the caveat that the confidence intervals do overlap with those from the RCT. Subject to the assumptions of our research design, these results indicate that there may be a substantial amount of bias from model misspecification. That is, the functional form assumptions that are encoded into both of these methods are contributing to a bias that substantially overstates the effect of RHC compared to the RCT results.

**TABLE 2:** The effect of RHC on mortality: Regression adjustment and IP weighting.

		Mortality
Regression adjustment	Point estimate	0.063
	95% Confidence interval	[0.037, 0.09]
Parametric G‐Formula	Point estimate	0.063
	95% Confidence interval	[0.036, 0.090]
IP weighting	Point estimate	0.061
	95% Confidence interval	[0.023, 0.099]

*Note*: Point estimates are differences in proportions who die by 6 months.

**FIGURE 2:**
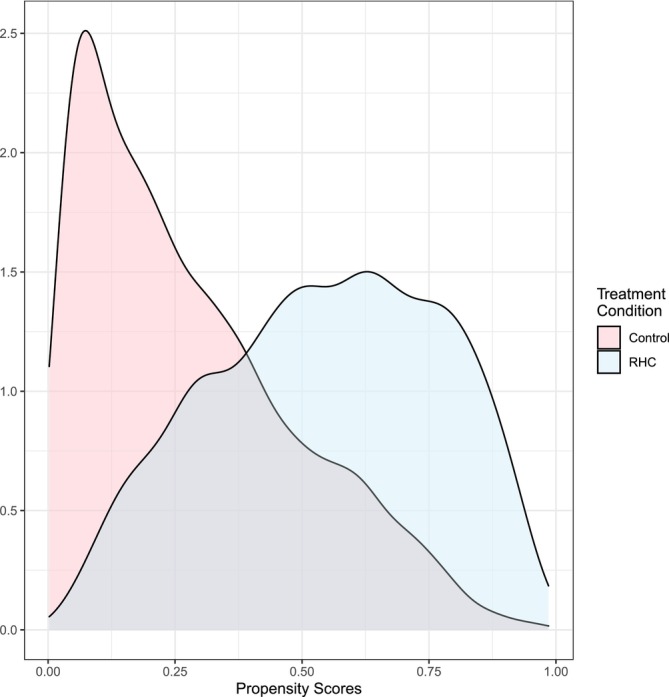
Distribution of propensity scores by treatment category.

One concern about applying regression and IPW within this case study is a lack of overlap in the distribution of baseline covariates across the treated and control groups. When there is a lack of overlap, there are often many control units that are far from the treated units in terms of the observed covariates. When this happens, it can lead to treatment effect estimates that are sensitive to statistical model, that is, model misspecification. In Figure 2, we plot the propensity scores by treatment condition. In this plot, we observe that there are many fewer control units that are close in terms of the propensity score to the treated units. The next method of statistical adjustment we apply is specifically designed to increase overlap between treated and control units.

Next, we report the results from using RC Balance matching. The match we used include several constraints to improve balance and increase overlap. First, we included a propensity score caliper. That is, while the match is based on generalized distances—using the Mahalanobis distance—we include a caliper on the propensity score to improve overlap. We iterated over the match until we found a caliper size that produced high levels of balance. We also included refined balance constraints to improve balance on the two disease categories variables, and the measures of type of insurance and race. These balance constraints and the propensity score caliper trimmed a substantial portion of the treated units. Recall that, in the data, there were 2184 RHC patients. After applying the balance constraints to the match, we were only able to retain 1547 RHC patients—thus changing the estimand. However, for this subset of treated patients, we were able to produce a highly balanced sample, whereby none of the standardized differences in means exceed 0.10. That is, none of the mean differences were greater than a tenth of a standard deviation. Moreover, several discrete covariates had nearly identical marginal distributions. We found that including higher numbers of treated units contributed to an imbalanced matched sample. Table 3 contains two sets of estimates. The first estimate is based on simply regressing the outcome on the treatment indicator using the matched data. The second estimate is based on regressing the outcome on the treatment indicator and all the baseline covariates for additional bias reduction. Both estimates indicate that RHC increases the risk of death by more than 7% points. These results indicate that the source of the model misspecification does not appear to be related to overlap. That is, the match ensured that we only estimated the results based on a highly comparable set of patients. Moreover, adding an outcome model did not result in any additional substantial changes in the treatment effect estimates.

**TABLE 3:** The effect of RHC on mortality: Matching.

		Mortality
Matching – RC balance	Point estimate	0.083
	95% Confidence interval	[0.051, 0.115]
Matching & regression adjust.	Point estimate	0.073
	95% Confidence interval	[0.042, 0.104]

*Note*: Point estimates are differences in proportions who die by 6‐months. Due to trimming of treated observations, estimand is no longer the ATT.

**FIGURE 3:**
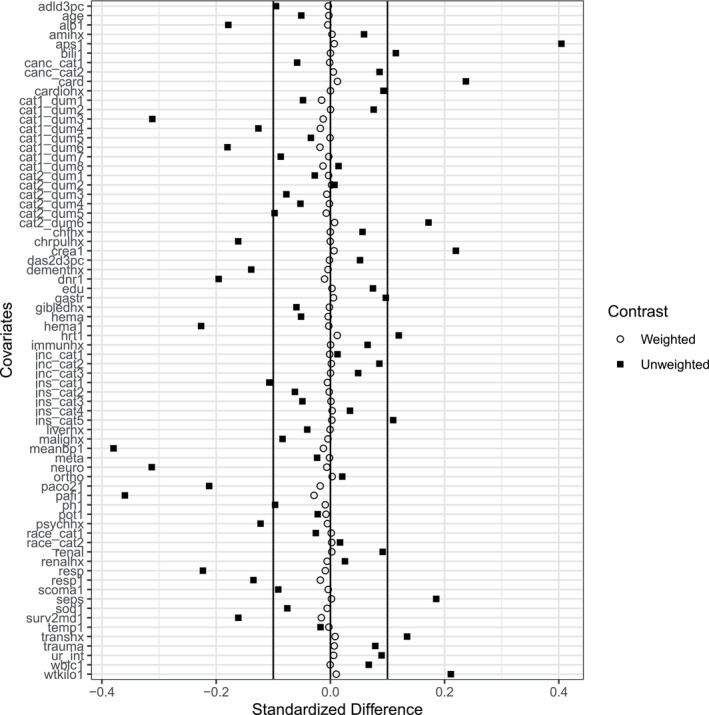
Balance plot for balancing weights. Imbalance measured via standardized difference.

Next, we implemented a more advanced form of weighting estimator that relies on balancing weights—weights that target specific balance constraints. First, we present a standard diagnostic for weights: balance statistics. Weighting methods should balance the observables, and we can visualize the extent to which that happens. Figure 3 contains a plot of the standardized differences—the difference in covariate means divided by the pooled treated and control standard deviation—before and after weighting. What is clear in the plot is that balancing weights produce a highly balanced sample.

Table 4 contains the results based on balancing weights both with and without additional outcome adjustment. Again, both estimates indicate that RHC increases the risk of mortality by over 6%. All four methods have returned estimates that are all highly comparable in terms of magnitude, but also appear to suffer from bias due to model misspecification. It is worth noting that thus far, we have not chosen to add additional interactions or nonlinear terms to any of the methods. Given the number of covariates, the process of adding the full possible set of interactions and nonlinear terms would be time consuming.

**TABLE 4:** The effect of RHC on mortality: Balancing weights.

		Mortality
Balancing weights	Point estimate	0.062
	95% Confidence interval	[0.038, 0.087]
Balancing weights & Regression adjust.	Point estimate	0.064
	95% Confidence interval	[0.043, 0.086]

*Note*: Point estimates are differences in proportions who die by 6‐months.

Next, we focus on DRML based methods of statistical adjustment. Of particular interest is whether these more flexible estimation methods based on ML can reduce model misspecification. Table 5 contains those results. Using GRF, we find that the treatment effect estimate is smaller, 2.6%, with confidence interval that includes zero. GRF, then, is the first method that moves the estimate closer to the experimental benchmark. In fact, the GRF estimate is nearly identical to the RCT estimate of 2.7%. Next, we applied the TMLE and SL approach. Notably, this collection of learners produces a point estimate that is farther from the experimental benchmark than the GRF estimate. Given how the close the GRF estimates are to the RCT benchmark, we re‐fit the TMLE and SL method only including a random forest. Now the estimate moves closer to the experimental benchmark. Interestingly, this behavior is similar to that found in Keele, OÃćâĆňâĎćNeill, and Grieve [76] where GRF methods outperformed the SL ensemble approach. The ability to include several ML methods in the SL approach may be unsuccessful in terms of reducing model misspecification.

**TABLE 5:** The effect of RHC on mortality: ML methods.

		Mortality
Random forest	Point estimate	0.026
	95% Confidence interval	[−0.001, 0.053]
TMLE & SL‐1	Point estimate	0.046
	95% Confidence interval	[0.025, 0.068]
TMLE & SL‐2	Point estimate	0.038
	95% Confidence interval	[0.019, 0.059]

*Note*: Point estimates are differences in proportions for Mortality. SL‐1: Learners: GLM, Random Forest, GAM, Lasso, and GBM. SL‐2: Learners: Random Forest.

To further understand the source of the model misspecification, we performed an additional exploratory analysis. In this secondary analysis, we split the sample into two random partitions. In the first partition, comprised of 25% of the data, we fit a random forest to model e(x). We used the random forest to identify key interactions. We then added these interactions to the specification for the balancing weight estimator applied to the second partition of the data. In this step, we balanced those interactions along with the main effects using balancing weights. We found that the balancing weights performed well in terms of balancing both the main effects and the interactions. We also added these interactions to the regression model used for outcome modeling. Table 6 contains the results from these additional analyses. First, we observe that balancing these interactions does little to reduce model misspecification: the reduction in mortality remains 5.7%. However, when these interactions are included in an outcome model with the balancing weights, we find that we can reduce model misspecification: the reduction in mortality is now 1.7%, quite close to the RCT estimate of 2.7%. This additional analysis demonstrates the value of ML methods in terms of reducing model misspecification by identifying key interactions between variables. Moreover, this demonstrates how for this more sophisticated use of balancing weights can act as a compliment or alternative to DRML approaches.

**TABLE 6:** The effect of RHC on mortality: Balancing weights combined with random forest.

		Mortality
Balancing weights	Point estimate	0.057
	95% Confidence interval	[0.027, 0.088]
Balancing weights & Regression adjust.	Point estimate	0.017
	95% Confidence interval	[−0.019, 0.052]

*Note*: Point estimates are differences in proportions for Mortality.

## 6 Discussion

This tutorial directly addresses a crucial challenge facing applied health researchers which is how to choose from amongst the multitude of available methods for statistical adjustment. Rather than prescribing a specific method, which may be contingent on the study circumstances, our tutorial offers a set of guiding principles to help analysts assess the robustness of results across types of methods that make different assumptions about model specification. We recommend that investigators apply outcome‐focused, treatment‐focused, and DR‐focused methods. Any disagreement between the three approaches is evidence that at least one of the methods is subject to model misspecification.

Next, our tutorial provides background on both traditional methods but also highly flexible ML methods. Recent work has developed the DRML framework which offers the best theoretical properties of any of the available options: nonparametric flexibility with optimal inferential properties. In the re‐analysis of the case study, we find that the NRS estimates from applying DRML methods were closest to those from the RCT benchmark reflecting the general advantage of ML methods in that they can flexibly include interactions between key variables. Hence, we recommend that studies consider applying DRML approaches for the main analysis.

Unfortunately, DRML methods may be impractical in many settings. DRML methods are relatively new and may be unavailable for many data configurations. For example, DRML methods aren't widely available in software for many kinds of survival analysis or longitudinal data applications. DRML software is generally confined to R, and can be difficult to implement with large sample sizes. For example, even when applied to the moderate sample size in our case study, the TMLE and SL fit with the full set of learners required over two hours of computing time. Our reanalysis and previous theoretical work suggest that in those settings where DRML methods are infeasible or undesirable, balancing weights are a reasonable alternative. Key advantages of balancing weights include: highly balanced treated and control distributions, easy inclusion of outcome models for a DR approach, and that with large sample sizes these approaches remain computationally efficient. As we demonstrated in the RHC application, one can also use ML methods to create a more flexible specification for balancing weights. This method could be combined with other methods such as matching. In general, analysts should be aware that when there are a larger number of baseline covariates, many of which are multi‐valued, the risk of model misspecification increases and the need grows for more flexible methods of estimation. Moreover, while regression modeling may be the most prone to misspecification bias, methods such as matching and weighting often do not protect against misspecification bias unless nonlinearities or appropriate interactions are included.

This tutorial focused on a selection of alternative approaches for reducing the risk of bias due to model misspecification in those settings when it is reasonable to assume that all relevant confounders have been measured prior to treatment assignment at a single timepoint. The general concern about selecting approaches to reduce the risk of bias from model mispecification applies more widely to other settings including instrumental variable designs, or those where major concerns are time‐varying confounding, transporting RCT estimates to target populations, or handling censoring or missing data. In conclusion, this tutorial provides a set of guiding principles to help analysts reduce the risk of bias from model misspecification when providing estimates of comparative effectiveness. All the methods discussed in this tutorial can be implemented in the open source general‐purpose software for R. In the appendix, we provide an overview of the code used for each analysis in this paper, with full replication materials (data and codes) available at https://github.com/ljk20/somanychoices.

## Conflicts of Interest

The authors declare no conflicts of interest.

## Data Availability

The data that support the findings of this study are openly available in Github at https://github.com/ljk20/choices.

## Appendix A Annotated Sample Code

Full replication files can be found at https://github.com/ljk20/somanychoices. The replication files include the data and scripts for R to generate every result in the paper. Note that these commands will not exactly replicate the results in the tables due to handling of missing values. For exact replication of tables, see the replication codes.

First, we do simple regression adjustment, we only output the results for the treatment effect.

Next, we implement IPW. First, we load a set of packages that we will use for a robust variance estimator.

Next, we include the code we used to implement matching. Note that here we include the code but do not execute the actual commands, which are somewhat time‐consuming. Please see the full replication materials for fully executable code.

Next, we include a balancing weights analysis. Again, we do not execute the code here.

Next, we run generalized random forests. The key function in this library takes the covariates as a matrix and not a data frame. As such, we process the data somewhat differently. In our experience, it is also useful to remove variable names that can cause errors.

Finally, we include a Superlearner example that uses an ensemble of ML methods. Here, the user selects the set of ML methods for fitting the nuisance functions.
